# Sustainable Eco-Friendly Synthesis of Gold Nanoparticles Anchored on Graphene Oxide: Influence of Reductant Concentration on Nanoparticle Morphology

**DOI:** 10.3390/ma18133003

**Published:** 2025-06-25

**Authors:** Mariano Palomba, Gianfranco Carotenuto, Maria Grazia Raucci, Antonio Ruotolo, Angela Longo

**Affiliations:** 1 Institute for Polymers, Composites, and Biomaterials, National Research Council, SS Napoli/Portici, 80125 Napoli, Italy; mariano.palomba@cnr.it (M.P.); giancaro@unina.it (G.C.); mariagrazia.raucci@cnr.it (M.G.R.); 2 Department of Engineering, College of Charleston, Charleston, SC 29424, USA; ruotoloa@cofc.edu

**Keywords:** gold nanoparticles, graphene oxide, green reducing agent, surface stabilizer

## Abstract

Gold nanoparticles (AuNPs) anchored on graphene oxide (GO) have had a significant interest for their unique optical, electrical, and catalytic properties. This study presents an eco-friendly and sustainable synthesis of AuNPs on GO sheets using L-ascorbic acid (L-aa) as a green reducing agent and polyvinylpyrrolidone (PVP) as a stabilizer. The effect of reductant concentration on nanoparticle morphology was systematically investigated using UV–Visible spectroscopy and transmission electron microscopy (TEM). Results indicate the formation of AuNPs anchored on GO sheets and that an increase in the L-aa amount leads to both an increase in nanoparticle size and a morphological transition from spherical to irregular structures. The simultaneous nucleation and growth processes result in the formation of multiple families of nanostructures, as confirmed by TEM analysis, which reveals two distinct size distributions. At higher L-aa concentrations, the nanoparticles shape evolves into irregular morphologies due to selective growth along a preferential facet. This approach not only enables precise control over AuNP size and shape but also aligns with green chemistry principles, making it a promising route for applications in plasmonics, sensors, and photothermal therapy.

## 1. Introduction

The sustainable synthesis of gold nanoparticles (AuNPs) anchored on graphene oxide (GO) sheets has garnered significant attention due to their combined functional properties [1] which hold promise for electronics [2], optoelectronics [2], catalysis [3], and particularly for biomedical applications [4].

AuNPs are highly efficient at absorbing light and converting it into heat, making them ideal for photothermal therapy, where near-infrared light is used to generate localized heat and destroy cancer cells [5]. In addition, AuNPs possess biocompatibility, antioxidant properties, and anti-inflammatory effects, which make them suitable for enhancing bone regeneration. The inclusion of GO provides mechanical strength, increased surface area, and bioactivity, all of which are beneficial in improving cellular interactions and tissue regeneration [6]. GO also acts as a biocompatible carrier and stabilizer for AuNPs, due to its large surface area, thereby enhancing their stability, dispersibility, and overall efficacy in photothermal therapy [7]. Moreover, GO absorbs near-infrared radiation, further contributing to heat generation and amplifying treatment effectiveness [8]. By controlling the morphology of nanoparticles, influenced by the reductant concentration, these particles can be optimized for use in biological systems.

When it comes to osteoporotic bone regeneration, AuNPs can promote the differentiation of stem cells into osteoblasts, stimulate bone matrix production, and enhance bone mineralization [9]. They can also facilitate the delivery of growth factors or drugs directly to the bone tissue, helping to repair damaged bone and regenerate osteoporotic defects. Moreover, the eco-friendly synthesis method ensures that these nanoparticles are safe and sustainable, reducing the risk of toxicity when used in clinical applications. Thus, these gold nanoparticle-functionalized graphene oxide composites can play a vital role in the development of advanced therapeutic strategies for bone regeneration, offering promising prospects for the treatment of osteoporosis and other bone-related diseases.

Furthermore, as mentioned, the integration of AuNPs and GO is revolutionizing electronics. Reduced graphene oxide (rGO) offers excellent electrical conductivity, which is further improved by the anchored AuNPs. These nanoparticles form conductive bridges between GO sheets, reducing electron scattering and enhancing composite performance [10]. The surface plasmon resonance (SPR) of AuNPs amplifies light–matter interactions, enhancing the efficiency of high-performance optoelectronic devices, such as photodetectors and solar cells, offering a unique combination of conductivity, flexibility, and sensitivity. In addition, applications range from flexible, transparent electronics to energy storage systems and wearable devices [1,11,12].

Nowadays, the control of the plasmonic absorption and morphology of AuNPs remains a critical focus for advancing next-generation technologies.

In addition, the growing attention towards sustainability emphasizes the use of eco-friendly materials, minimizing toxic chemicals, and developing methods of synthesis reducing energy consumption, maximizing efficiency [13,14]. A widely used method for synthesizing AuNPs anchored on GO is simultaneous reduction in an aqueous solution. Typically, a gold precursor (i.e., HAuCl_4_) is mixed with a GO suspension, and a green reducing agent, like plant extracts, microorganisms [15], or rose water [16], etc., is added to reduce Au^3+^ to Au^0^, forming AuNPs directly on GO sheets. This approach replaces toxic chemicals like hydrazine and sodium borohydride with green alternatives, adhering to the principles of green chemistry. Among green reductants, L-ascorbic acid (L-aa) stands out for its effectiveness [17,18,19]. L-aa facilitates the controlled synthesis of gold and silver nanoparticles and efficiently reduces GO to rGO, yielding materials with enhanced electrical conductivity. Key advantages of using L-ascorbic acid include the following:

- Environmentally friendly and non-toxic properties.
- High efficiency in reducing metal ions, enabling the synthesis of nanoparticles with controlled size and morphology.
- Effective reduction of GO to rGO with improved properties.
- Low risk of introducing impurities, as L-aa contains only carbon, oxygen, and hydrogen atoms.

This approach not only leverages the advanced properties of AuNPs and GO but also aligns with green chemistry principles, ensuring a sustainable and efficient synthesis process.

Here, a simple method to obtain colloid solutions of AuNPs anchored on GO by reduction of HAuCl_4_ using L-aa is presented. The stability of colloid solutions is ensured by the presence of polyvinylpyrrolidone (PVP) as a stabilizer [20]. In addition, the influence of reductant concentration on nanoparticle morphology was analyzed through UV–Visible spectroscopy and transmission electron microscopy (TEM).

## 2. Materials and Methods

### 2.1. Materials

The precursors used for the synthesis of the AuNPs/graphene colloidal solution were as follows: (i) graphene oxide (GO) nanosheets as the graphene-based support; (ii) tetrachloroauric acid (HAuCl_4_, Sigma-Aldrich, St. Louis, MO, USA, 99.9%) as the metal nanoparticle precursor; (iii) L-ascorbic acid (L-aa, C_6_H_8_O_6_, Sigma-Aldrich, SSt. Louis, MO, USA, reagent grade) as a green reducing agent; and (iv) poly(N-vinylpyrrolidone) (PVP, Sigma-Aldrich, St. Louis, MO, USA, Mw = 10,000 a.m.u.) as a protective agent. 

For the synthesis of GO nanosheets, the following reagents were used without further purification: sulfuric acid (H_2_SO_4_, Sigma-Aldrich, St. Louis, MO, USA, 99.999%), potassium nitrate (KNO_3_, Sigma-Aldrich, >99.0%), potassium permanganate (KMnO_4_, Sigma-Aldrich, St. Louis, MO, USA, >99.0%), hydrogen peroxide (H_2_O_2_, Sigma-Aldrich, St. Louis, MO, USA, 30%), and anhydrous ethanol (C_2_H_6_O, Carlo Erba, Milano, Italy, ACS reagent). 

Additionally, distilled water was used for all experiments. 

### 2.2. GO Nanosheet Synthesis

GO nanosheets were synthesized using a modified Hummers’ method applied to graphite nanoplatelets [21]. A mixture of graphite nanoplatelets and KNO_3_ in H_2_SO_4_ was stirred for a few minutes in an ice bath. KMnO_4_ was then slowly added under stirring to prevent the temperature from exceeding 20 °C. The mixture was subsequently stirred for 1 h at 35 °C. A color change from dark purplish green to dark brown confirmed the oxidation of the nanoplatelets. 

To stop the oxidation process, distilled water was gradually added to the solution, followed by the addition of a diluted aqueous H_2_O_2_ solution (≈4% *v*/*v*). The resulting GO nanosheets were separated from the reaction mixture by filtration and purified through successive cycles of centrifugation, ultrasonication, and re-dispersion in water until a pH of 5–6 was achieved. Further details on this synthesis method can be found in previous publications [18] and references therein. 

### 2.3. Synthesis of AuNPs/Graphene Colloid Solutions

The preparation of AuNPs/graphene colloid solutions involved three steps. Firstly, a solution was prepared by dissolving 1 g of PVP into 10 g of distillate water. To ensure complete dissolution of PVP in water and obtain a stable solution, the mixture was stirred continuously for 48 h at room temperature [22]. After that, 1 mL of aqueous solution of GO (40% in weight) was added. This main solution contains PVP, as a passivating agent, and GO, as a graphene-like substrate for gold nanoparticle deposition. A second solution was freshly prepared by dissolving 12.5 mg of tetrachloroauric acid in distilled water at room temperature. This solution containing the metallic precursor was added to the main solution under continuous stirring. Then, a freshly aqueous solution of L-aa, as a reducing agent, was quickly injected under stirring and made to react for 15 min.

To investigate the effect of L-ascorbic acid (L-aa) on the size and morphology of gold nanoparticles (AuNPs), the weight ratio between the metallic acid and the reducing agent varied, starting from stoichiometric conditions. 

For this, the balanced chemical equation between HAuCl_4_ and L-aa is as follows:

2 HAuCl_4_ + 3 C_6_H_8_O_6_ = 2 Au + 3 C_6_H_6_O_6_ + 8 HCl (1)

In stoichiometric conditions, where molar ratio R = 2/3 and for a fixed amount of gold produced (i.e., 12 mg of HAuCl_4_), the corresponding weight ratio of the reactants is 1.29. This value was used as the starting point, from which the amount of L-aa was progressively increased to explore its effect on nanoparticle formation. 

The amount of L-aa added influenced the nucleation process, which in turn affected the size and morphology of the obtained AuNPs and promoted the partial reduction of GO. Table 1 summarizes the amounts used for the preparation of the different samples. 

**Figure 1:**
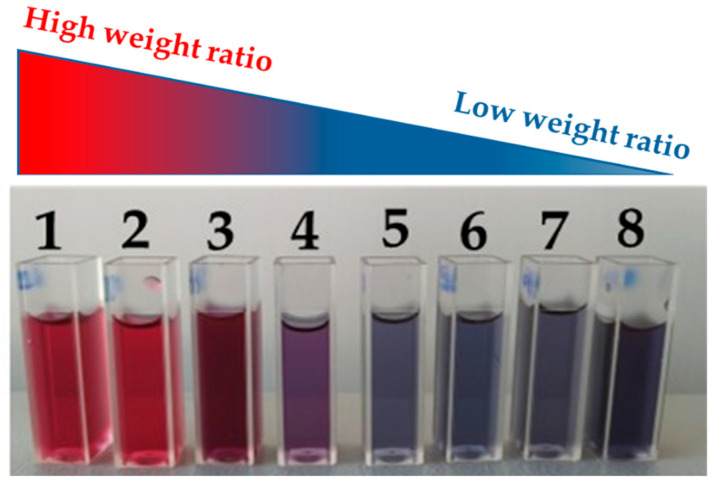
Different samples of AuNPs/graphene colloid solution obtained by varying the weight ratio of HAuCl_4_/L-aa.

As visible in Figure 1, eight different samples were obtained, with a color shift from red to blue, by varying the weight ratio, R, from 1.29 to 0.51. It is well known that this typical optical property of metal nanoparticles, named Surface Plasmon Resonance (SPR), is strictly dependent on the shape and the size of the nanoparticles.

### 2.4. Instrumentations

The spectra of precursors and the SPR of colloid solutions were acquired with a Perkin-Elmer Lambda 850 spectrophotometer (PerkinElmer, Waltham, MA, USA), at room temperature (25 °C) between 200 nm and 900 nm with a scan speed of 800 nm/min and a read interval of 1 nm. For measurements, an aliquot of the sample was placed into a 1 cm path length quartz cuvette in the sample light path, while the distilled water was placed into a quartz cuvette in the reference light path.

Qualitative and quantitative elemental analysis measurements were performed by scanning electron microscope (SEM) (FEI Quanta 200 FEG, Hillsboro, OR, USA) equipped with an Oxford Inca Energy Dispersive X-ray (EDX) microanalyzer (Inca Oxford 250, High Wycombe, UK).

The synthesized colloid solutions were morphologically characterized by TEM to investigate the sheet-like morphology of rGO, and the formation of AuNPs and their size and dispersion patterns. A few drops of each sample were air-dried on a copper grid coated with carbon film for TEM analysis. The measurements were carried out by an FEI Tecnai G2 Spirit TWIN (Philips, Amsterdam, The Netherlands) microscope, operating at 120 kV and equipped with a LaB6 filament. The sizes of AuNPs from TEM images were analyzed using the National Institutes of Health (NIH) Image J1.48i. This software is an open-source image processing software designed to analyze multidimensional scientific images, such as TEM micrographs. Measurements needed to analyze the changes in AuNP morphology were constructed using the Feret diameter. This diameter is a measure of an object’s size along a specified direction. In general, it can be defined as the distance between the two parallel planes restricting the object perpendicular to that direction. This measure is used in the analysis of particle sizes, for example, in microscopy, where it is applied to projections of a three-dimensional (3D) object on a 2D plane [23].

The effect of the L-aa amount on sample morphology was analyzed by comparing optical results with morphological characterization.

## 3. Results and Discussion

This section is divided into two subparagraphs, providing a detailed description of the experimental results, their interpretation, and the corresponding discussion. To ensure a clearer and more concise presentation, selected experimental results from only three samples, i.e., sample 1, sample 4, and sample 8, are presented, as they exhibit distinct and specific properties.

### 3.1. Optical Characterization

The optical spectra of all precursors were acquired and a comparison of these spectra with those of the obtained samples confirms the complete conversion of the precursors through the reduction process.

**Figure 2:**
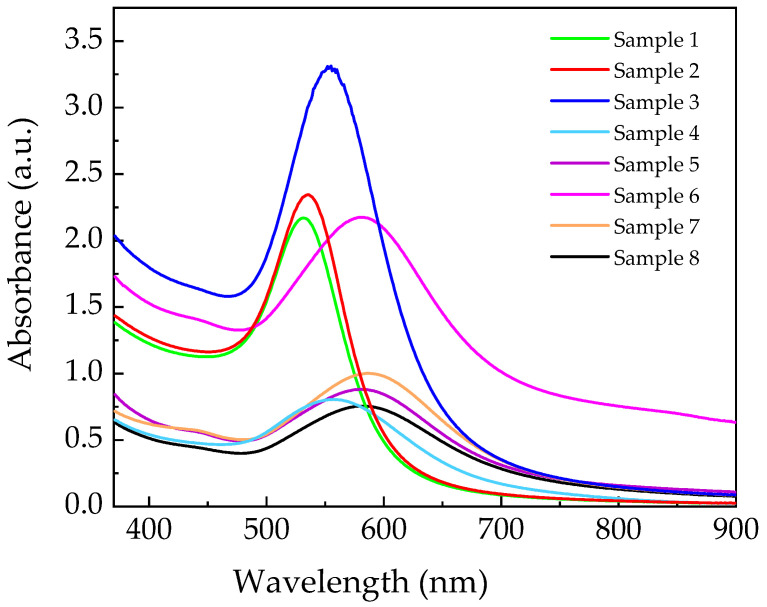
UV–Visible spectra of AuNPs/graphene colloid solution obtained by varying the weight ratio of HAuCl_4_/L-aa.

Figure 2 illustrates UV–Visible spectra of the obtained colloid solution of AuNPs/graphene obtained using different weight ratio, R, from 1.29 to 0.51 characterized by a presence of a typical SPR peak. As previously mentioned, the SPR peak position (λ_m_) of Au nanoparticles is strictly dependent on size and shape [24]. As shown by the experimental spectra, the wavelength (λ_m_) of the SPR peak shifts to a higher wavelength, indicating that the samples contain Au nanoparticles with variations in size and shape.

**Figure 3:**
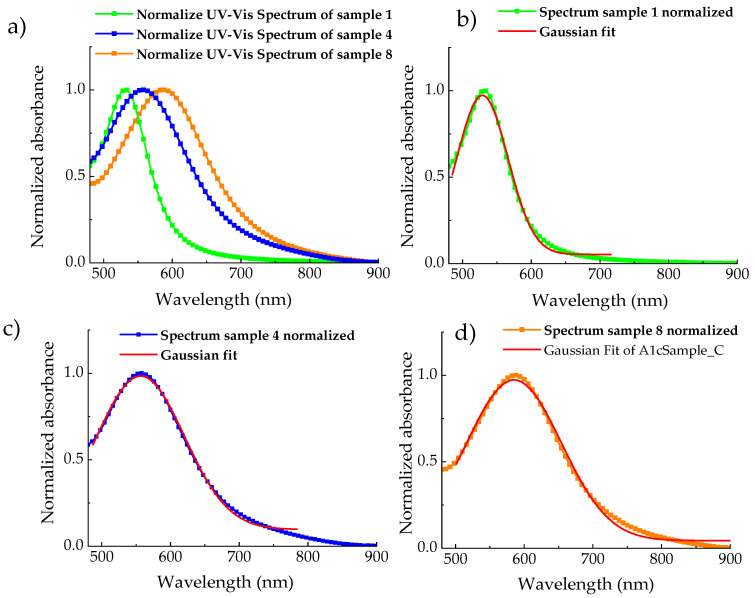
UV–Visible spectra of AuNPs/graphene colloid solution of sample 1, sample 4, and sample 8 normalized (**a**), and the fit of experimental data by Gaussian curve of AuNPs/graphene colloid solution of sample 1, sample 4, and sample 8 (**b**), (**c**), and (**d**), respectively.

All spectra were normalized to maximum values in the range from 450 to 900 nm and fitted with a Gaussian curve to better evaluate the λ_m_ position. In Figure 3, for greater clarity, only spectra of sample 1, sample 4, and sample 8 are shown.

**Figure 4:**
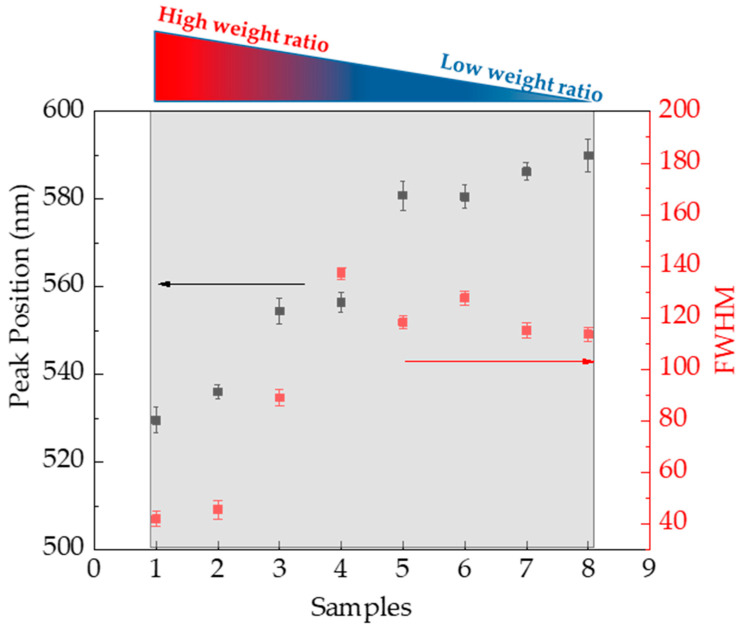
Peak position of SPR, λ_m_, and the full width at half maximum (FWHM) obtained by UV–Visible spectra of all AuNPs/graphene colloid solutions.

**Figure 5:**
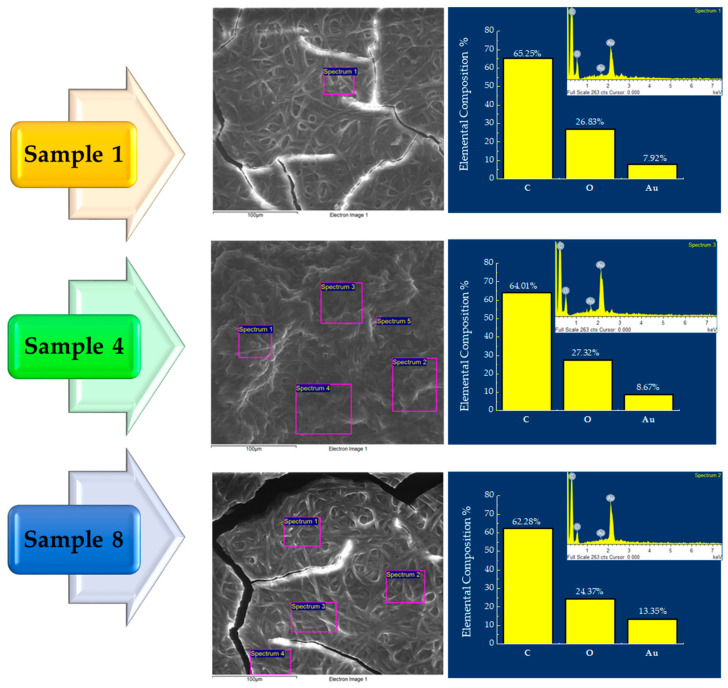
SEM micrographs and corresponding EDS elemental analysis of samples 1, 4, and 8. Each image indicates the area where EDS was performed. The elemental composition is presented as bar charts showing weight percentages of C, O, and Au. Inset: representative EDS spectra for each sample.

Accordingly, Figure 4 summarizes the behavior of the peak position of SPR, λ_m_, and the full width at half maximum (FWHM), obtained through the Gaussian fit of the optical spectra. As the weight ratio R decreases from sample 1 to sample 8, λ_m_ shifts to higher wavelengths, from 530 nm to 590 nm (see black squares and line in Figure 5). Similarly, the FWHM increases from 25 to 140 as R decreases from sample 1 to sample 4, then stabilizes around 130 (see red circles and line in Figure 5). These trends indicate that small, monodisperse Au nanoparticles were obtained with a stoichiometric amount of L-aa, whereas larger nanoparticles with irregular shapes formed at higher concentrations.

The main observation is thus that, as R decreases, the main SPR band red-shifts and becomes broader, resulting in a band/shoulder corresponding to the nanostructure asperities mode becoming more intense. Such changes in the optical response can be easily justified in terms of the morphology of the particles.

### 3.2. Morphological and Structural Characterization

EDS analysis was performed on the samples to determine the chemical composition and identify potential impurities. SEM micrographs of sample 1, sample 4, and sample 8 at 10,000× magnification and the EDS analysis, are shown in Figure 5, along with histograms displaying the element composition percentages. SEM images acquired at low magnification were used solely to indicate the region where EDS elemental analysis was performed. These images do not aim to provide morphological information. The element Au constitutes, on average, around 9 wt% (percentage in weight) of the total composition for all samples. Additionally, due to the presence of PVP and GO, the amounts of carbon and oxygen remain relatively constant across all samples, at approximately 60 wt% and 25 wt%, respectively. The inset in Figure 5 displays the elemental spectra obtained for all samples.

**Figure 6:**
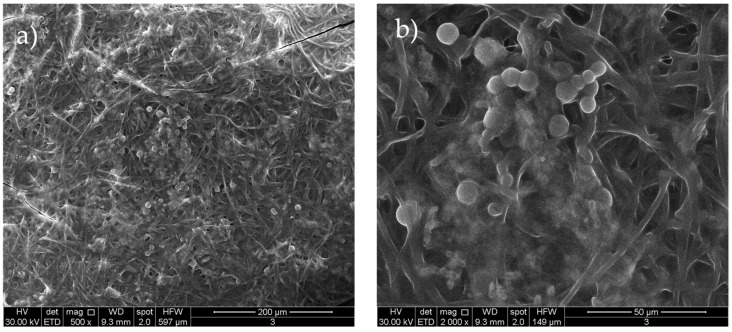
SEM of AuNPs distributed in a stabilizing PVP of typical sample 3 at two different magnifications (**a**,**b**).

Representative SEM images of sample 3 (see Figure 6) are shown to highlight the composite nature of the hybrid system. The SEM images clearly reveal the presence of spherical nanoparticles, attributable to AuNPs, embedded within a polymeric matrix. Unfortunately, due to their similar morphology and low contrast in SEM, the GO sheets cannot be distinguished from the surrounding PVP matrix.

**Figure 7:**
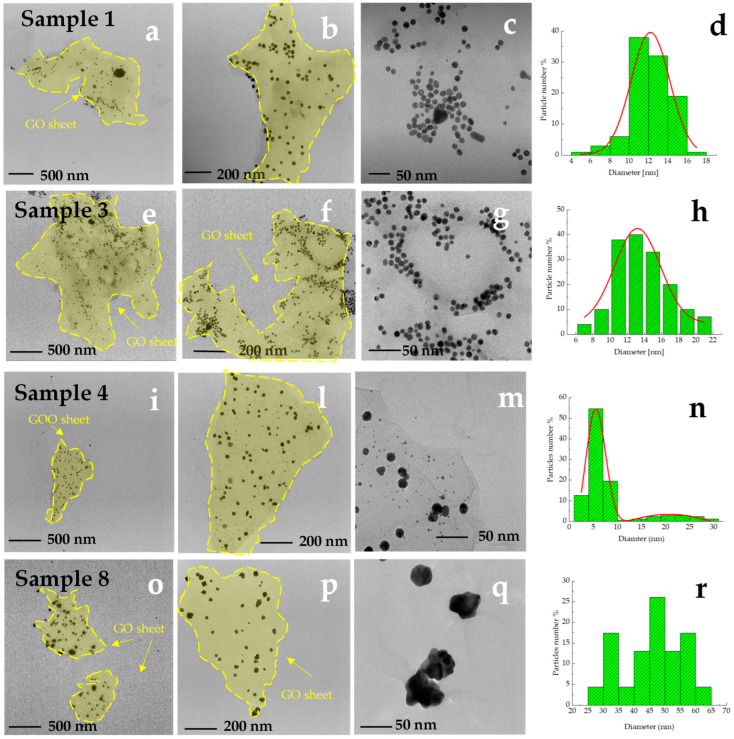
TEM images of samples 1 (**a**–**c**), 3 (**e**–**g**), 4 (**i**,**l**,**m**), and 8 (**o**–**q**) at three magnification levels showing the morphological evolution of AuNPs as a function of the [L-aa]/[HAuCl_4_] ratio. The histograms (**d**,**h**,**n**,**r**) show Feret diameter distributions for each sample. In low magnification panels, e.g., (**a**,**e**), semi-transparent sheet-like structures corresponding to graphene oxide (GO) indicated by yellow line are visible as substrates anchoring the AuNPs.

For this purpose, the TEM analysis was carried out. Figure 7 shows TEM images of sample 1, sample 3, sample 4, and sample 8. The micrographs clearly display the successful synthesis of AuNPs anchored on graphene oxide sheets (indicated in figure by yellow line), and reveal that the morphology of AuNPs anchored to the surface differs significantly and is strongly related to decrease in weight ratio R from 1.29 to 0.51. As shown by the micrographs at different magnifications of sample 1 (see Figure 7a–c) at high weight ratio, the AuNPs are well-anchored on the GO surface and are nearly uniform and homogenous in their spherical shape and size. In addition, the histograms (see Figure 7d) show that the average size of the nanoparticles is 11 nm, with a relatively narrow distribution. The micrographs of sample 3 (Figure 7e–g) show nanoparticles that are anchored on the GO sheet, predominantly spherical, and comparable in size and distribution to those in sample 1 (Figure 7a–c) and sample 2 (data not shown). A comparison of the Feret diameter distributions (see histograms in Figure 7d,h) shows a similar Gaussian-like profile in samples 1 and 3, with mean values centered around ~11 nm and ~13 nm, respectively. These results confirm that, in both cases, the nucleation and growth processes occur under comparable kinetic regimes, resulting in spherical nanoparticles with narrow size distributions. These observations suggest that no significant morphological changes occur between samples 1 and 3, supporting the idea that nanoparticle aggregation and morphology alteration begin to be prominent only from sample 4 onward. Decreasing R ratio, the sample 4 micrographs (see Figure 7i,l,m) reveal the formation, on GO sheet, of two different AuNPs classes. It is possible to identify a class of uniform and spherical nanoparticles characterized by the smallest size, and a second group characterized by irregular shape and biggest size. The distribution shows two average sizes centered at 6 nm and 22 nm (see Figure 7n). In the case of lowest R, the sample 8 micrographs, see Figure 7o–q, clearly reveal a formation of AuNPs anchored on the GO sheet characterized by irregular shapes and multiple protrusions radiating from a central core. This structure results from anisotropic growth mechanisms triggered by the high L-aa concentration, which alters both nucleation kinetics and the capping efficiency of PVP. The number of particles is reduced, consistent with fewer nucleation events and extensive growth, possibly driven by Ostwald ripening. The broad Feret diameter distribution (Figure 7r), spanning from ~30 to 65 nm, reflects the heterogeneous population of branched nanostructures formed under these conditions.

In other words, at lower magnification (e.g., panels Figure 7a,e,i,p), the presence of thin, wrinkled GO sheets (see yellow line in Figure 7) can be clearly identified as semi-transparent layers supporting the AuNPs. These sheets are typical of exfoliated GO and provide evidence that the nanoparticles are anchored on the GO surface, not freely dispersed. Although the polymeric matrix of PVP is a fundamental stabilizer in the colloid system, it is not detectable in TEM images due to its low electron density and high transparency to the electron beam. Consequently, the uniform background observed in several micrographs does not indicate an absence of GO but rather the non-observable nature of PVP in these imaging conditions. In addition, significant changes in morphology and size distribution appear only from sample 4 onward, where bimodal size populations and irregular shapes become evident. As shown in Figure 7c, AuNPs in Sample 1 exhibit a predominantly spherical morphology, with narrow size distribution and good dispersion on GO sheets. In contrast, AuNPs in Sample 7 display (see Figure 7m) a branched and irregular structure, consistent with uncontrolled growth and preferential facet development. These observations confirm the morphological evolution induced by L-aa concentration, as also reflected in the shift and broadening of the size distribution histograms.

This marks the onset of morphology transition due to increased L-aa concentration and simultaneous growth mechanisms, as also confirmed by the UV-Vis spectral red-shift and FWHM broadening.

These results confirm the eco-friendly effectiveness of L-aa as a reducing agent for both HAuCl_4_ and highlight the role of PVP as a passivating and stabilizing agent. PVP is well known for its ability to enhance the dispersion of nanoparticles in aqueous systems and is frequently employed in combination with graphene-based materials to improve colloidal stability [22]. Furthermore, it has been reported to assist in transporting gold nanoparticles into the interlaminar regions of GO, while simultaneously stabilizing their structure [25]. In this configuration, PVP not only prevents nanoparticle aggregation, but also promotes effective anchoring of AuNPs onto the GO surface. This structural arrangement has been widely described in the literature for its role in improving both nanoparticle distribution and overall composite stability [20,25].

At low concentrations of L-aa, the shape of the AuNPs is thermodynamically controlled, leading to spherical structures due to the adsorption and stabilizing effect of PVP. In this case, the formation of the particles follows a two-step process. The first step is nucleation, during which the average particle radius is less than a few nanometers. Subsequently, nanoparticle growth is believed to occur through either Ostwald ripening or coalescence. With increasing L-aa concentration, smaller spherical AuNP seeds form due to a reduction in nanoparticle size. In this scenario, nucleation and growth occur simultaneously, resulting in multiple families of nanostructures. This phenomenon is particularly evident in the TEM micrographs, which reveal two distinct families with different size distributions. As the L-aa concentration continues to increase, this aggregation process further evolves, ultimately leading to the formation of irregular AuNPs. Additionally, a notable decrease in particle number is observed, accompanied by an increase in particle size, which can be directly attributed to Ostwald ripening.

These morphological evolutions are consistent with UV–Visible observations, particularly the progressive red-shift and broadening of the SPR band (Figure 5), indicating increased size and shape anisotropy. Such trends are attributed to the combined effect of L-aa concentration on nucleation kinetics, partial reduction of GO, and local pH variations, which modulate the balance between thermodynamic and kinetic growth regimes.

In other words, beyond the observed effects of the reducing agent concentration on nanoparticle size and morphology, our investigation reveals that increasing the concentration of L-ascorbic acid induces a slight decrease in the pH of the reaction medium. This pH shift plays a critical role in modulating the nucleation and growth mechanisms of AuNPs. Under near-neutral conditions, the efficient passivation provided by PVP leads to the formation of small, monodisperse, and predominantly spherical nanoparticles. However, when higher amounts of L-ascorbic acid are used, the resulting lower pH can diminish the capping efficiency of PVP. This change promotes more rapid nucleation and anisotropic growth, giving rise to irregular or irregular, branched nanoparticle morphologies. Therefore, careful control of both the reducing agent concentration and pH is essential for fine-tuning the structural properties of the nanoparticles, ultimately broadening their applicability in plasmonic, sensors, and photothermal therapy.

## 4. Conclusions

In conclusion, a sustainability and green chemistry approach is presented. The synthesis of AuNPs anchored on GO is performed using an environmentally friendly reducing agent (L-ascorbic acid), replacing toxic chemicals like hydrazine and sodium borohydride. In addition, enhanced stability and better dispersibility are obtained thanks to the use of polyvinylpyrrolidone (PVP) as a stabilizer that improves the stability of colloidal solutions and prevents nanoparticle aggregation. The study systematically investigates how the concentration of the reducing agent influences nanoparticle size and shape, providing valuable insights for tuning material properties.

The UV–Visible spectroscopy, TEM, and SEM-EDS analyses confirm the successful synthesis, composition, and morphology of the hybrid structures. Depending on the reducing agent concentration, the synthesized AuNPs exhibit spherical, irregular, and star-like morphologies, which significantly influence their potential applications in plasmonic, sensors, and photothermal therapy. While the morphological and optical results confirm the successful formation of AuNPs/GO composites stabilized by PVP, additional structural and chemical analyses such as XRD, XPS, and TGA would further validate the incorporation and interaction of each component. These characterizations are planned for future studies to deepen the understanding of the hybrid system. The method is simple, cost-effective, and scalable, making it suitable for industrial applications in nanoelectronics and sustainable materials development.

## Figures and Tables

**Table 1:** The amounts of the reducing agent and the weight ratio AuHCl_4_/L-aa for the preparation of the AuNPs/graphene hybrid structures.

Name	[HAuCl_4_] mg	[L-aa]	Ratio [HAuCl_4_]/[L-aa]
Sample 1	12.52	9.72	1.29
Sample 2	12.36	12.75	0.96
Sample 3	12.49	14.66	0.85
Sample 4	12.59	16.72	0.75
Sample 5	12.10	18.77	0.64
Sample 6	12.84	20.22	0.60
Sample 7	12.31	22.66	0.54
Sample 8	12.57	24.52	0.51

## Data Availability

The data presented in this study are available on request from the corresponding author.
